# Endoplasmic Reticulum Stress and Its Role in Homeostasis and Immunity of Central and Peripheral Neurons

**DOI:** 10.3389/fimmu.2022.859703

**Published:** 2022-04-27

**Authors:** Caroline Perner, Elke Krüger

**Affiliations:** ^1^Center for Immunology and Inflammatory Diseases, Division of Rheumatology, Allergy and Immunology, Massachusetts General Hospital, Harvard Medical School, Boston, MA, United States; ^2^Department of Neurology, Universitätsmedizin Greifswald, Greifswald, Germany; ^3^Institute of Medical Biochemistry and Molecular Biology, Universitätsmedizin Greifswald, Greifswald, Germany

**Keywords:** ER-stress, unfolded and integrated stress response, innate immunity, central nervous system, peripheral nervous system

## Abstract

Neuronal cells are specialists for rapid transfer and translation of information. Their electrical properties relay on a precise regulation of ion levels while their communication *via* neurotransmitters and neuropeptides depends on a high protein and lipid turnover. The endoplasmic Reticulum (ER) is fundamental to provide these necessary requirements for optimal neuronal function. Accumulation of misfolded proteins in the ER lumen, reactive oxygen species and exogenous stimulants like infections, chemical irritants and mechanical harm can induce ER stress, often followed by an ER stress response to reinstate cellular homeostasis. Imbedded between glial-, endothelial-, stromal-, and immune cells neurons are constantly in communication and influenced by their local environment. In this review, we discuss concepts of tissue homeostasis and innate immunity in the central and peripheral nervous system with a focus on its influence on ER stress, the unfolded protein response, and implications for health and disease.

## Peripheral Sensory Neurons Regulate Host Defense and Tissue Homeostasis

Cell and tissue homeostasis are the foundation of a physiological and healthy organism. Homeostasis means to balance and properly regulate all required components and interactions by actively maintaining certain quantitative characteristics of the system, known as regulated variables, within a desired range (1). This implies a proper osmoregulation, regulation of ion-levels and membrane potentials, pH regulation, glucose and lipid metabolism, protein synthesis, protein folding and degradation on a cellular level, hormone production, thermoregulation, enzyme secretion, transmitter release and host defense on tissue and organismic level. Multicellular organisms have developed a complex machinery, that allows and requires the interaction of multiple cell types to prevent the body from harm, and heal tissue damage. Inflammatory responses represent such a mechanism often required to prevent disruptions in homeostasis and rebuilt it if interruptions occur.

On an organismic level, innate and adaptive immunity are the main drivers for host defense. The innate immune system is evolutionally older and consists of myeloid cells (macrophages, monocytes, microglia, dendritic cells, neutrophils, basophils, eosinophils, mast cells) and NK-cells as well as the complement system. Pathogens invading the organism get recognized through conserved surface structures that are broadly shared by pathogens (glycans, glycoconjugates, lipopolysaccharides (LPS), endotoxins found on cell membranes from gram negative bacteria) and distinguishable from host molecules and therefore called pathogen-associated molecular patterns (PAMPs) that bind to pattern recognition receptors (PRRs) expressed by innate immune cells such as toll-like receptors (TLRs), C-Type lectin receptors (CLRs), NOD-like receptors (NLR & NLRPs) RIG-I-like receptors (RLRs) (2). This allows the cellular innate immune response to start within minutes, reflected by phagocytosis of the invading pathogens or the release of mediators (granzyme, histamine, bradykinin, serotonin, leukotrienes, prostaglandins), cytokines, and chemokines that further promote the elimination of the pathogen by recruiting other immune cells in or directly harm the pathogen (3, 4). Besides the recognition of pathogens, the immune system also recognizes molecules released due to tissue damage (heat-shock proteins, high mobility group protein B 1 (HMGB1), hyaluronan fragments, ATP, uric acid, heparin sulfate, DNA, RNA), called damage associated molecular patterns (DAMPs). DAMPs are endogenous danger signals and can activate innate immune cells trough PRRs to respond to non-infectious “sterile” tissue damage. Cytosolic nucleic acids are sensed by a number of cytosolic nucleic acid receptors such as cyclic-GMP-AMP-synthase (cGAS) and stimulator of interferon (IFN) genes (STING) for DNA or by RIG-like receptors for RNA. These nucleic receptor in turn activate the phosphorylation of transcription factors like IFN regulatory factor 3 (IRF3) by TANK-binding kinase 1 (TBK1) important for the transcription of type I IFN like IFN-α or -β. These proinflammatory cytokines along with a couple of IFN-stimulated molecules can serve as a physical barrier against the spread of the infection but also promote tissue healing (5). But there is also an increasing body of evidence pointing to PRR activation through mitochondrial DNA that will drive IFN-driven inflammation contributing to disease formation (6).

Moreover, we and others discovered recently that there is another cell type involved in the recognition of harmful invaders like bacteria, fungi, and allergens or toxins. Peripheral sensory neurons have a big impact in the initiation of the local inflammation and contribute to the activation of innate and adaptive immune cells (7–10). By sharing many PRRs with innate immune cells and expressing other G-protein coupled receptors (GPCRs) for the recognition of harmful substances like toxins and allergens, they are functioning as a first sensor of tissue damage. Within seconds, these sensory neurons are alarming the host of a potential harm through perception of pain or itch. This is often followed by a neurological reflex arc. Examples are lifting the hand from a hot plate or scratching to remove insects or parasites on the skin, induction of sickness and the initiation of vomiting to remove toxic food intake before it can do more harm to just list some reflexes where the neuronal system acts to defend the host. But besides the neurological implication of the sensed harm by sensory neurons, they also actively participate in initiating inflammation and regulating the immune response. Upon sensing potential harm they react by the immediate release of certain neuropeptides into the tissue where the damage was sensed, but also, and this is the important difference, into the neighboring tissue that gets innervated by other branches of the same sensory neuron (9). This so-called anticipatory immunity allows for an immediate warning of the neighboring tissue and induces inflammation through the release of neuropeptides like calcitonin gene-related peptide (CGRP), Substance P, vasoaktives intestinales peptid (VIP), Pituitary adenylate cyclase-activating polypeptide (PACAP) and Chemokine-like protein TAFA-4 (TAFA-4) (7, 11–13). The release pattern is depended on the pathogen, toxin and tissue damage. Sensory neurons and their neuropeptides are thereby contributing to all aspects of inflammation as defined by Celsus: rubor, calor, tumor, dolor and change of function. Therefore neuropeptides induce vasodilatation, extravasation and function as chemoattractant for immune cells to migrate into the tissue (14). Neuropeptides can induce either a proinflammatory or anti-inflammatory immune response, and promote tissue healing through their respective receptors expressed by immune cells, endothelial cells, and glia cells (15, 16). Thus, small dysregulations in sensory neuron and immune cell function can influence the immune response required to defend properly from a harmful stimulus and rebuild tissue homeostasis as the immune feedback regulation between pro-inflammatory signals for pathogen eradication and anti-inflammation for tissue healing can easily be disrupted.

## Unfolded Protein and Integrated Stress Responses - Between Cell Homeostasis – Cell Defense – and Cell Death

**Figure 1:**
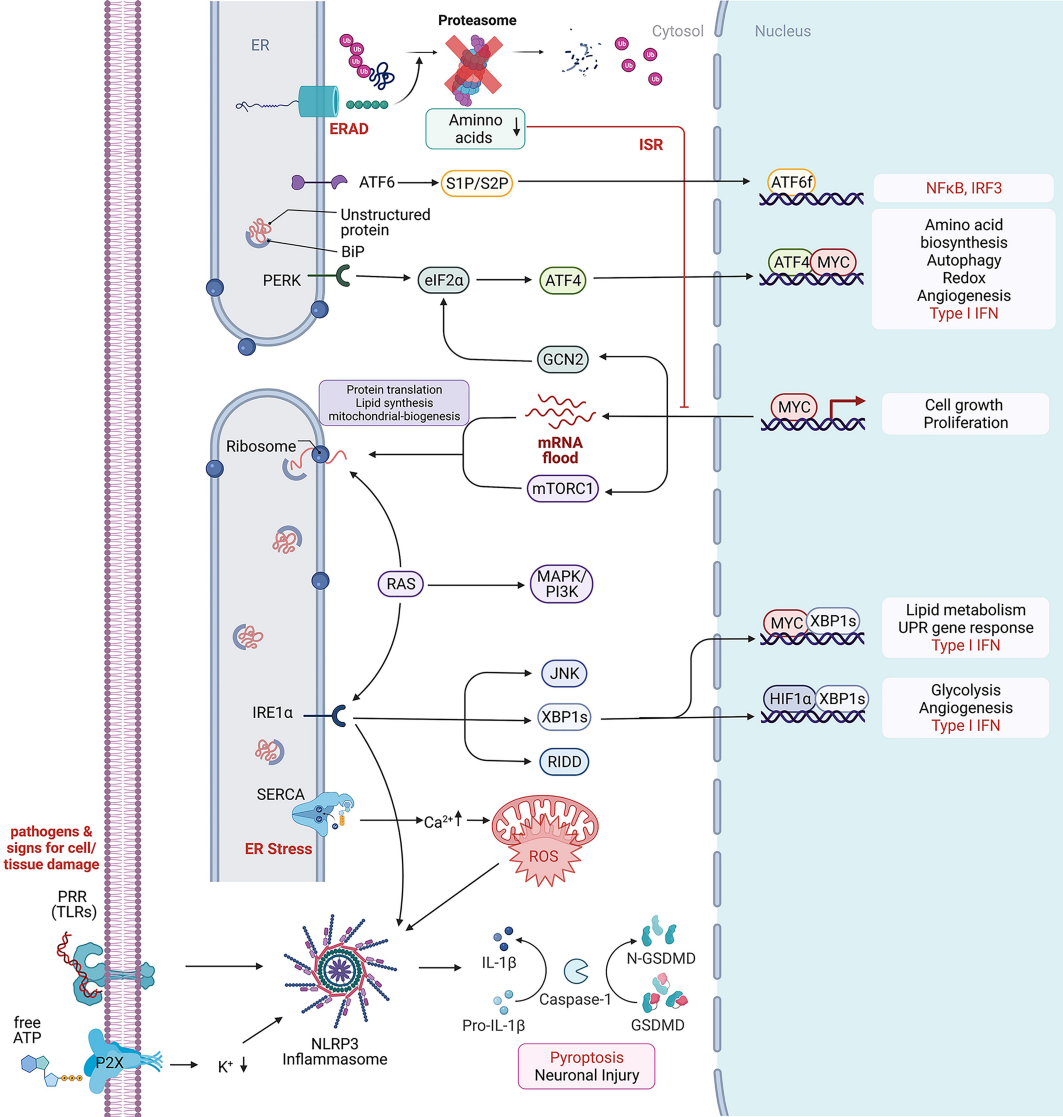
Cellular stress response mechanism. Depicted are intracellular signaling pathways involved in the stress response against extracellular and intracellular stressors. From top to bottom: Endoplasmic-reticulum-associated protein degradation (ERAD) recognizes misfolded proteins of the endoplasmic reticulum (ER) and induces their ubiquitinylation for protein degradation through the proteasome. Low cytosolic amino acid levels can inhibit the proteasomal degradation through the proteasome and further inhibit cell growth and proliferation. Unfolded or misfolded proteins within the ER activate BiP, a major chaperone in the initiation of the unfolded protein response, that further interacts with (ATF6) and the protein kinase RNA-like endoplasmic reticulum kinase (PERK) to induce the UPR, leading to ATF6f and ATF4 dependent transcriptional modification. In rapidly proliferating cells, MYC dependent transcription enriches the cytosol with mRNAs waiting for proper translation on ribosomes. Protein folding mistakes can be recognized by the inositol-requiring enzyme 1 α (IRE1α), that *via* splicing of X-box binding protein 1 (XBP1) induces transcriptional modification and thereby lipid and glycose metabolism. The RNase domain of IRE1 mediates the cleavage of multiple RNAs in a process known as regulated IRE1-dependent decay (RIDD) (19). c-Jun N-terminal kinase (JNK) activation may be linked to ER stress by IRE1 and autophagy induction after ER stress relies on the IRE1-JNK pathway (20). The ER is highly involved in calcium homeostasis by acting as a calcium storage. Ion-pumps like the sarco/endoplasmic reticulum Ca2+-ATPase (SERCA) are actively transporting calcium between the cytosol and ER lumen. High cytosolic calcium concentration can increase the production of reactive oxygen species (ROS) that further are able to promote NLR family pyrin domain containing 3 (NLRP3) inflammasome activation which is usually activated by exogenous pathogens, recognized by their RNA/DNA on pattern recognition receptors (PRR) like Toll-like receptors (TLR) and the involved tissue damage products like ATP that can be recognized by purinergic receptors like P2R. Created with BioRender.com.

We discussed above how inflammatory processes are involved in restoring tissue homeostasis in response to infection or injury. On a cellular level, inflammation can occur without infection or damage, but as a response for a disruption in cellular homeostasis (1, 17, 18) Intracellular dysregulation, such as endoplasmic reticulum (ER), mitochondrial or osmotic stress reaching a level that normal homeostatic regulators cannot control, are known to trigger different response mechanism related to inflammation. ER stress is often caused by an increase in misfolded proteins as well as through dysregulations in ER calcium homeostasis or lipid membrane integrity (**Figure 1**). These stressors are able to induce the unfolded protein response (UPR) that increases transcription of proteins that help with protein processing such as chaperons, the ER-associated degradation system (ERAD), proteins important for lipid metabolism, energy homeostasis and cell proliferation. If cellular homeostasis cannot be rebuilt by the UPR, it can induce apoptosis (21, 22). The UPR stress sensors inositol-requiring protein 1α (IRE1α), protein kinase RNA-like endoplasmic reticulum (ER) kinase (PERK) and activating transcription factor 6 (ATF6) are ER membrane proteins. They transduce information about the protein folding status to the cytosol and nucleus and induce transcription of UPR target genes *via* splicing of X-box binding protein 1 (XBP1), phosphorylation of eukaryotic translation initiation factor 2α (eIF2α) that can attenuate general protein synthesis and allows translation of ATF4 mRNA to ATF4, which controls the transcription of genes involved in autophagy, apoptosis, amino acid metabolism and antioxidant responses (22). ATF6 is a leucin zipper transcription factor, which is transported to the Golgi apparatus under ER stress conditions where it gets processed *via* S2P and S1P to release its cytosolic domain fragment and upregulate the gene expression of ERAD and XBP1 components. Thus, the UPR has a major role in preserving correct protein folding, regulate protein degradation, cell metabolism and control of cell organelles thereby protects from cellular death through autophagy (23). Interestingly, by protein quality control of cytokine production, modulation of cytokine transcription factors and cytokine receptor expression, the UPR is also directly involved in inflammation and immunity (24). Activated *via* TLRs and PRR, ER stress is reported to increase the expression of Interleukine-23 (IL-23) in myeloid cells in response to the intracellular bacterium Chlamydia trachomatis (25). There is further evidence that ER stress and UPR primes cells to respond to innate immune stimuli by activating the IRF3 transcription factor that synergistically augments early type I IFNs and other genes involved in innate immunity (26–29).

Interestingly, there is a big overlap with the diseases associated with ER stress and the UPR (30). Both pathways are important in cellular inflammation and stress response and connected *via* reactive oxygen species, mitochondrial dysfunction, a disbalance in ion levels, cytokines and Angiotensin II (31). Furthermore, ER stress can independently of the UPR response activate the NLRP3 inflammasome in a Voltage-dependent anion selective channel 1 (VDAC1) dependent manner (32). VDAC1 is located in the outer mitochondrial membrane where it allows ATP to diffuse out of the mitochondria into the cytosol. It is also important for the volume regulation and acts in scaffolding proteins such as hexokinase that are important for metabolic regulation (33). VDAC1 overexpression is therefore known to be associated with Alzheimer´s disease and Parkinson’s disease (34, 35). While inflammasome activation and IL-1β secretion is important in the defense against bacteria, type I IFNs are important for the defense against viral infection sensed through PRRs sensitive to double stranded RNA and DNA. There is increasing evidence for an association of the pathways associated with type I IFNs with neuroinflammation and neurodegenerative diseases just recently reviewed by Fryer A et. al (36). Therefore, the cGAS-STING or RIG-MAVS pathways can also be involved in the initiation of an inflammatory response through the production of type I IFN in the absence of pathogens. Another important cellular response that can be activated through double stranded RNA as well as by IFNs is the integrated stress response (ISR). Similar to the UPR, the ISR is involved in the inhibition of global translation to decrease viral mRNA translation and viral protein assembly, while increasing the translation of selective mRNAs recognized through internal ribosome entry sites (IRESs) or upstream open reading frames (uORFs), such as ATF4. The ISR can be activated through four stress activated kinases, i.e. PERK, protein kinase R (PKR), general control nonderepressible 2 (GCN2), or heme-regulated inhibitor (HRI). Proteotoxic stress by ER stress, misfolded proteins, oxidative stress and proteasome inhibition activate both the ISR and UPR intercrossing *via* PERK. Viral infection/dsDNA and associated IFNs activate the ISR through PKR. Amino acid deprivation through proteasomal dysregulation, RNA virus infection and UV irradiation are activating the ISR through GCN2, and low heme, heat shock proteins and mitochondrial dysfunction are associated with ISR activation through HRI (37–39). Not surprisingly, there are multiple reports about the ISR to be involved in neurodegeneration as reviewed by Martinez NW et al. (40) and Bond S et al. (37). More recently, proteotoxic stress has been correlated to ER stress and UPR/ISR activation in rare proteasomopathies. This disease spectrum caused by inborn defects of the proteasome includes neuronal manifestation and is linked to dysregulation of type I IFN signaling (41, 42). Mechanistically, PKR serves as an innate immune sensor for proteotoxic stress *via* cytoplasmic accumulation of IL-24 (43).

UPR and ISR are involved in sensing homeostatic disturbances and are important to restore cellular and tissue homeostasis. As they are intertwined through their stimuli, signaling proteins and through their successful degradation and negative regulation through proteasomal degradation, it is not surprising that they are influencing each other. Disruptions in one of them are likely to induce the other stress response in time and if not regulated properly, they will contribute to cell death rather than restore homeostasis. In this vicious cycle of cell decline due to a dysregulation in cellular stress response, there can be multiple reasons that lead to imbalance, starting a process of a chronic stress response and inflammation. Genetic mutations, metabolic stress due to an unhealthy lifestyle and multiple mechanism associated with aging are known to be highly associated. One rarely discussed cause though, is the accumulation of certain infections during lifetime.

In the last years researchers provided new evidence for a possible causation of viral infection for the development of Alzheimer´s disease (AD) (44–46). In fact, there is evidence that Amyloid beta (Aß) -oligomers bind herpesvirus surface glycoproteins, accelerating ß-amyloid deposition, which is leading to a protective viral entrapment (46). Nevertheless, the accelerated Aß - deposition may induce an increased inflammatory response with a following Aß - pathology and contribute to the continuous and chronic activation of the UPR and inflammasome cellular stress response pathways, initiating the development of neurodegenerative diseases years before the first symptoms occur. Especially now with new RNA-vaccination methods developing for viruses that are known to be difficult to immunize against like severe acute respiratory syndrome coronavirus 2 (SARS-CoV-2) (47), human immunodeficiency viruses (HIV) (48) and ZIKA virus (49), thinking about vaccinations for viruses likely associated with neurodegenerative diseases like the herpes virus family might be a way to protect from the vicious cycle of UPR, ISR and inflammasome response started through the viral infection. Furthermore, there are more and more clinical trials using inflammasome, ISR and UPR modification as a promising treatment against inflammatory and neurodegenerative diseases (**Table 1**). In this review, we will further provide an overview about the immune homeostasis (tissue and cellular homeostasis) in central and peripheral neurons, describing the connections to associated neurological diseases and possible treatment strategies.

**Table 1:** Treatments in clinical trials.

Drug/ Company	Rationale/preclinical studies	Target	Clinical trial
**Inzolemid/Inflazome Dublin, Ireland (acquired by Roche in 2020)**	-NLRP3 plays a role in diseases with chronic inflammation like Cryopyrin-associated periodic syndrome (CAPS) (50) and neurodegenerative diseases (51) -NLRP3 is a receptor for Aβ, mediating immune response in microglia (52) -Deletion of NLRP3 in APP/PS1 mice diminishes Aβ deposition, synapse loss and memory deficits (53) -Loss of NLRP3 in human Tau expressing mice prevents tau tangle formation (54) -related NLRP3 inhibitor MCC950 prevented inflammasome activation *via* α- synuclein in a model of Parkinson´s disease reducing neuronal loss and promoted (55) clearance of Aβ and cognitive function in APP/PS1 mice (56, 57)	NLRP3 Inflammasome	-Completion Phase 1 clinical trial march 2020 in healthy adults and patients with Cryopyrin-associated periodic syndrome (CAPS) Dublin, Ireland (acquired by Roche in 2020) -Phase 2 dose finding trial ongoing -prioritizing on CAPS, PD, AD and ALS patients
**NT-0167/NodThera Cambridge, UK; Seattle; and Boston**		Selective NLRP3 Inflammasome inhibitor	Phase 1 clinical trial completed in healthy volunteers -treatment of diseases driven by chronic inflammation (Liver and lung fibrosis, neurodegenerative diseases)
**Tauroursodeoxycholic Acid (TUDCA; TURSO)/Humanitas Mirasole SpA**	-has been used for centuries in Asian medicine -natural occurring bile acid -neuroprotective function (58) - reduced amyloid plaques and improved cognition in APP/PS1 mice (59) -improved pathology and behavior in mouse models of PD, HD, and ALS (60)	- inhibits mitochondria-mediated apoptosis and the formation of reactive oxygen species, and blocks apoptosis caused by ER stress	Phase 3 clinical trial in ALS (Amyotrophic Lateral Sclerosis) ongoing (2022) Phase 2 clinical trial (completed) slowed deterioration on the ALS Functional Rating Scale (ALSFRS-R) compared with placebo (61)
**sodium phenylbutyrate (PB)**	- treatment resulted in fewer plaques and better performance in a spatial memory task in a model of AD (APPswePS1delta9) (62) - significantly suppressed neuronal cell death caused by ER stress through chaperone activitiy (63)	- histone deacetylase inhibitor and chemical chaperone	Phase 1& 2 for Amyotrophic Lateral Sclerosis (ALS) (64) completed
**AMX0035/** **Amylyx Pharmaceuticals Inc**.	-combination of sodium phenylbutyrate (PB) and taurursodiol (TUDCA;TURSO)	- optimized to address both the toxic, unfolded proteins in the endoplasmic reticulum and the mitochondria associated oxidative stress associated that occur in neurodegenerative diseases	Amyotrophic Lateral Sclerosis (Phase 2) CENTAUR completed 2019 (65), Alzheimer’s Disease PEGASUS (Phase 2), results expected 2021
**Trazodone**	-in preclinical studies of neurodegenerative disease, prolonged overactivation of PERK/eIF2a-P signaling is followed by sustained attenuation of protein synthesis, leading to memory impairment and neuronal loss -Re-establishing translation rates by inhibition of eIF2a-P activity, genetically or pharmacologically, improves memory and prevents neurodegeneration and extends survival (66)	- reduces ATF4 levels - reverses eIF2α-P mediated translational attenuation (67) - blocker of the post-synaptic serotonin (5-HT) receptors 5-HT2A and 5-HT2C and inhibitor of presynaptic 5-HT reuptake transporters (68)	Phase 2 for painful diabetic neuropathy (69) completed
**Guanabenz**	- modulates protein synthesis by the activation of translational factors, preventing misfolded protein accumulation and ER overload (70) - promoting translational recovery in hippocampi of prion-infected mice is neuroprotective (71)	- binds to a regulatory subunit of protein phosphatase 1, PPP1R15A/GADD34 →selectively inhibits endoplasmic reticulum stress-induced eIF2a-phosphatase, allowing misfolded protein clearance, reduces neuronal death	Phase 2 in Amyotrophic lateral sclerosis (ALS) trial finished (72) Phase 1 for Multiple Sclerosis, trial finished. NCT02423083

## Physiological and Anatomical Features of the ER in Neurons – Implications for the Development of Neuropathology

**Figure 2:**
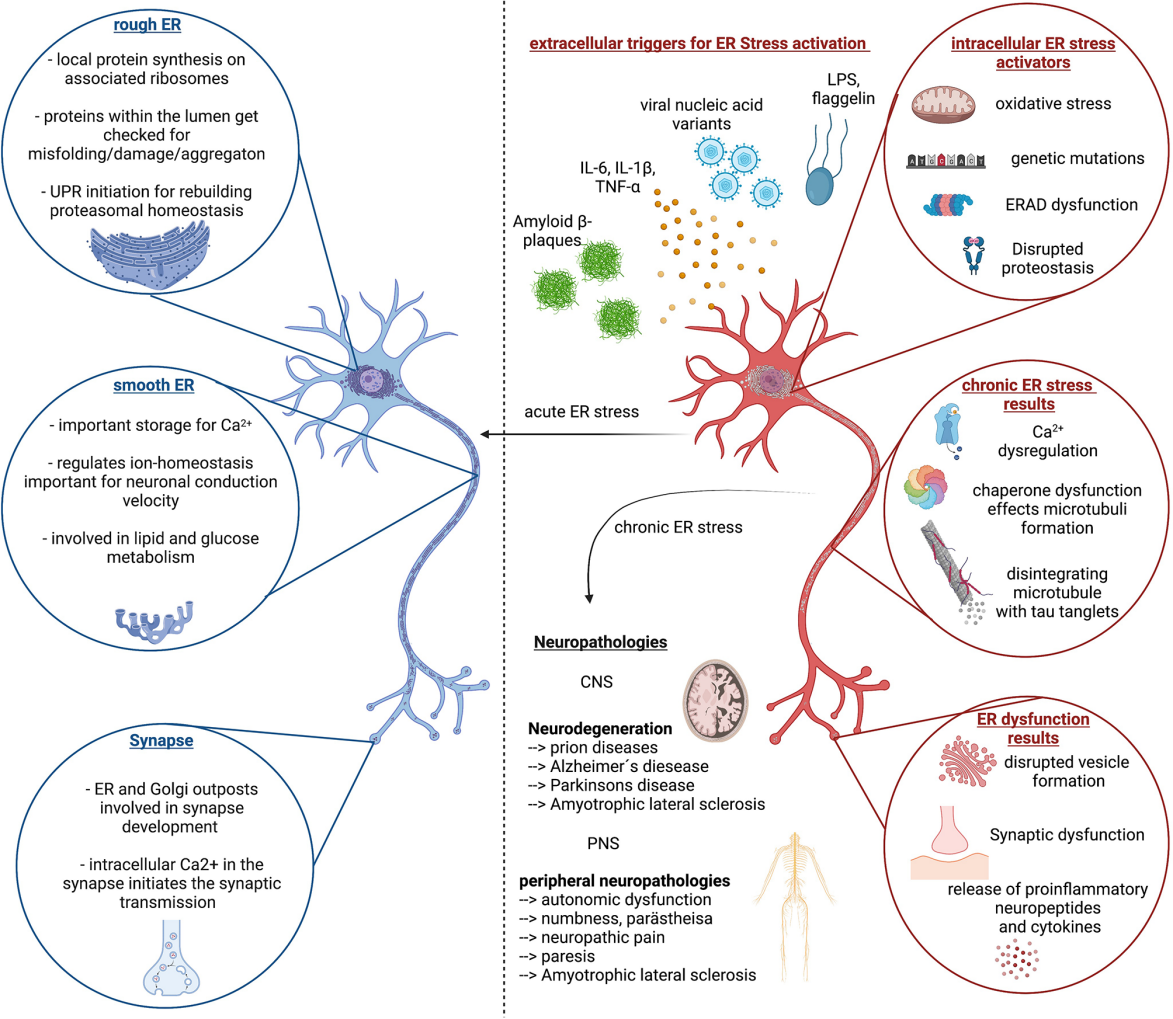
ER stress response in homeostasis and disease. Left (blue color) side depicts important function of the Endoplasmic reticulum (ER) in neuronal cells and highlights the importance of proper ER function for neuronal function, which is reinstated by the unfolded protein response (UPR) and proper regulation of proteasomal degradation and synthesis. The right side (red color) depicts the extra- and intracellular stressors that can lead to chronic ER stress, associated with continues disruption of neuronal cell homeostasis followed by dysfunction and decline. This circulus vicious is a foundation of multiple neurological diseases of the CNS and PNS of which the most common ones are listed here. Created with BioRender.com.

The ER is the largest organelle in the cytoplasm of eukaryotic cells. It is composed of a dynamic network of stacked membrane sheets connected with helicoidal motifs (73) with an inner lumen that is connected to the nuclear envelope (**Figure 2**). The ER that surrounds the nucleus is called rough ER as it contains densely packed ribosomes for protein synthesis on its surface. The proteins are transported from the cytosol into the ER lumen where they get folded and further modified by chaperons and folding enzymes, undergo quality control and get transported to their destination. Proteins that do not achieve a functional conformation within the ER are targeted by the ER quality control and enter the ERAD pathway and thus proteasomal degradation (74). Neurons belong to the biggest cells as their axons can reach over 1m in humans and even more in bigger animals. Therefore, the neuron has to produce a lot of essential proteins. As more protein synthesis is required, as more rough ER is located in the soma and dendrites of the neuron, where it can be seen as Nissl bodies. As the ribosomes are rich in RNA, they can be stained with basic dye. The axon hillock and axon do not contain rough, but smooth ER. The smooth ER with only a few ribosomes consists of connected cylindric tubules and interconnected cisternae. The tubules are involved in lipid biogenesis and glucose metabolism while the cisternae are functioning as calcium storage (75). Phospholipids which are the major lipid component of membranes get biochemically modified and transported together with proteins to an ER region in close juxtaposition to the Golgi apparatus, the ER-Golgi intermediate compartment ERGIC.

The ER functions as Ca^2+^ storage, keeping a concentration between 100-800µM. The typical cytosolic Ca^2+^ concentration is supposed to be around 100nM. In comparison, extracellular space has a Ca^2+^ concentration of around 2mM. If a cell gets activated through G-protein coupled receptors, phospholipase C (PLC) gets stimulated to cleave phosphatidylinositol 4,5 bisphosphate (PIP2) into diacyl-glycerol (DAG) and IP3, which can then bind to IP3R on the ER membrane leading to Ca^2+^ release from the ER lumen into the cytosol and transient increase in intracellular Ca^2+^levels (76). Ca^2+^ gets pumped back into the ER through the sarcoplasmic reticular calcium ATPases (SERCA) and can additionally enter the cell trough voltage- or ligand gated calcium channels from the extracellular space. The most important function and characteristic of neurons compared to other cell types is the excitability, the transport of information through action potentials as a result of a change in membrane potential. Therefore, it is of utmost importance for the neuron to regulate its ion levels and transfer ions as fast as possible to the right location. In the specific architecture of neurons the ER lumen is connected from the soma, through the whole axon to the synapsis, and thereby allows for fast shifts of ions, but also the transport of required neuropeptides and neurotransmitters for cell communication and signal transduction (77). Signal transmission across synapses can be performed through electric synapses with the transfer of electric signals through gap junctions or chemical synapses where neurotransmitters and neuropeptides get released as messengers. Respective specialized receptors on the post synaptic cell can bind the transmitters and peptides and forward the information through inhibitory or excitatory signals. Reuptake and recycling of neurotransmitters and neuropeptides ensures restorage, enabling the rapid frequency of synaptic transmission if necessary (78).

The ER is therefore a central organelle in neuron physiology. Disruptions of the ER functions are likely to cause dysfunction of neurons through a malfunctioning in protein and lipid biosynthesis for neurotransmitter and neuropeptide vesicles, and a disbalance in calcium levels necessary for the initiation of action potentials and signal transmission through synapses (**Figure 2**). In sum, ER stress is likely the consequence in different disease mechanism and can contribute positively and negatively to disease pathology for neuropathies and neuronal decline associated with many central and peripheral neurological diseases (79, 80).

## Immunity of the CNS

**Figure 3:**
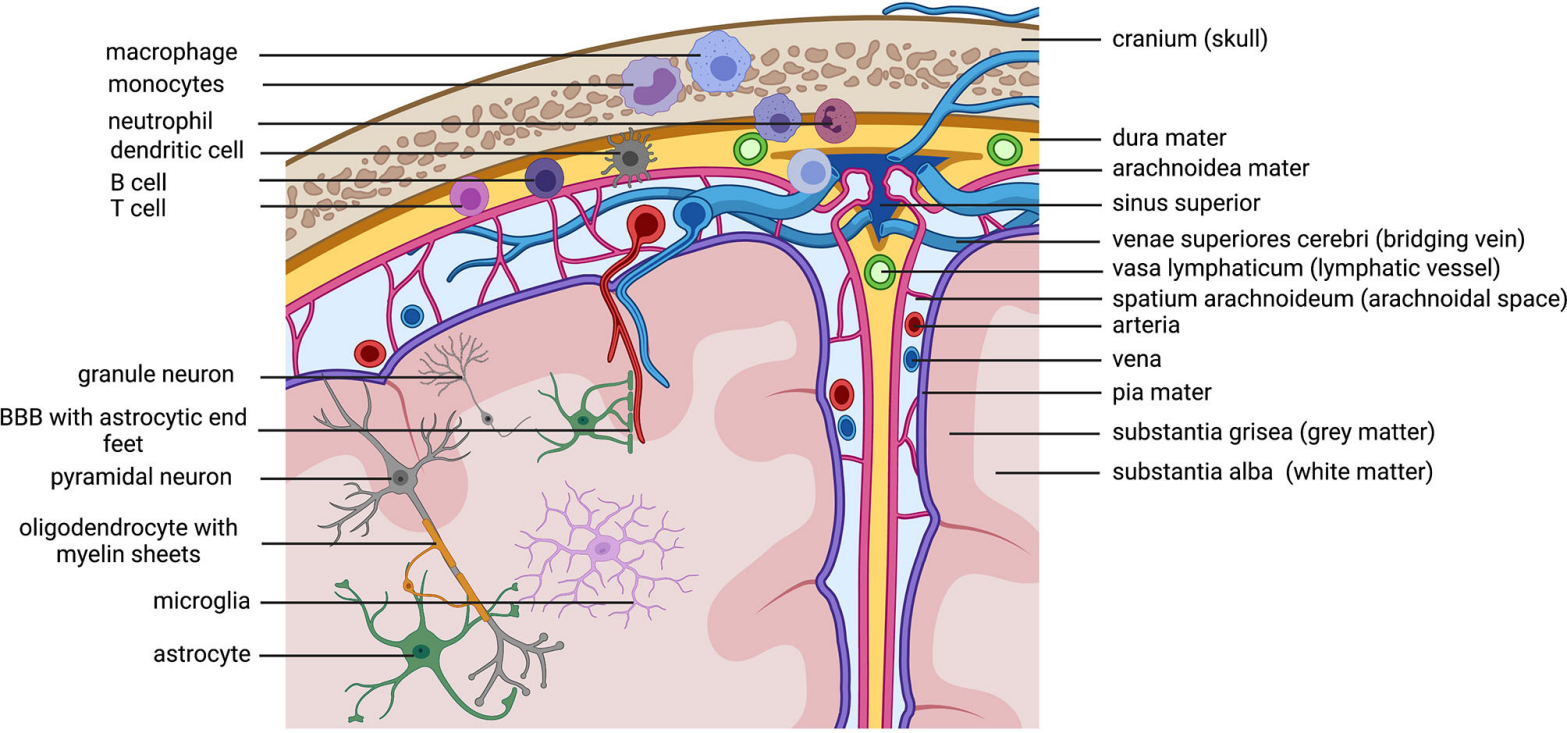
Immune cells patrolling the CNS. Macro anatomical structures important for the brain on the right side The brain is protected by the skull and the meninges. Within the thick dura mater are lymphatic vessels that allow immune cells located within the stroma of the dura mater to drain to the cervical lymphnodes (not shown in the figure). The arachnoidal space is filled with liquor (cerebrospinal fluid= CSF) that flows through the brain and contains and discards cerebral debris. Antigens within the CSF can get presented to patrolling immune cells in the dura mater close to the sinuses. Lymphocytes located in the dura are known to secrete cytokines that can reach the cerebral parenchyma and influence neuronal function. Microanatomical structures /cells on theleft side.Cave! The depiction has no correct scale, the cells are depicted to big. This is only a scheme to annotate involved cell types. Because of the scale the location of cells is not perfectly correct (neuronal cell bodies are always in the grey matter, microglia are also in the grey matter). Important to note is the location of immune cells in the dura mater. Myeloid cells get directly recruited from the bone marrow of the skull. Created with BioRender.com.

The central nervous system (CNS) contains of the brain and the spinal cord. It is surrounded by three layers of meninges, the very thin pia mater that covers the parenchyma and follows supplying arteries, the arachnoid mater, that consist of many spider like columns that build the arachnoid space that is filled with cerebrospinal fluid (CSF) and the outer, very thick and strong dura mater that is connected to the skull and vertebrate bones, builds compartments through the falx cerebri and accommodates the venous sinuses. The meninges offer protection of the CNS parenchyma as does the CSF, that functions as a shock absorber in the arachnoid space and it provides buoyancy, thereby reducing the overall brain weight to protect it from damage. Besides these physical implications for the CNS, the CSF and the meninges are actively contributing to CNS immunity. Other than in the periphery, the CNS is under common, non-inflammatory conditions isolated from blood circulating immune cells and most chemicals through the blood brain barrier (BBB). The BBB is composed of endothelial cells connected through tight junctions, followed by multiple layers of smooth muscle cells and finally large astrocyte (glial) end-feet. Many years there was the prominent concept of the immune-privilege of the brain, pointing to studies, where antigens (pathogens or allografts) implanted only into the brain could not induce a systemic immune response, which was different, if the immune system was pre-primed to the same antigen in the periphery before (81, 82). It was assumed, that this is due to a lack in lymphatics draining the brain from debris and antigens. Indeed, recent studies shed light on the mystery about the lymphatic drainage of the CNS and its impact for CNS immunity and neuronal function which is in detail reviewed by KA Lima, J Rustenhoven and J Kipnis (83). In fact, there are all kinds of innate and adaptive immune cells present in the meninges, especially in locations close to the dural sinuses (**Figure 3**). Myeloid cells are the dominating cell type and they are recruited directly from the skull bone marrow (84). But multiple studies point especially to a huge impact of cytokines released by lymphocytes in the meninges that can reach the brain parenchyma and directly influence behavior (85, 86). Thereby, meningeal immunity contributes actively through cytokines to CNS homeostasis and neuronal function. Studies with immune compromised mice shows behavioral cognitive deficits which could be rescued through transfer of CD4+ but not CD8+ T cells (87–91). T cells specific to CNS antigens were also shown to contribute to spatial learning, memory and neurogenesis (92). Depletion of CD4+ T cells was therefore shown to reduce hippocampal neurogenesis accompanied by impaired reversal learning in the Morris water maze (93). Indeed, there is evidence that meningeal T cells are supporting learning and memory through the expression of the key cytokine IL-4. Studies revealed a proinflammatory phenotype of meningeal myeloid cells in IL4(-/-) mice that was associated with cognitive impairment and could be reversed through transplantation of wild-type T cells (94, 95). Meningeal γδ T cells expressing high levels of the chemokine receptor CXC chemokine receptor 6 (CXCR6) are seeding meninges shortly after birth. Their release of IL-17a by these cells was shown to be correlated with anxiety-like behavior in mice and was partially dependent on T cell receptor engagement and commensal­ derived signals. IL-17a receptor is therefore expressed in cortical glutamatergic neurons under steady state and its genetic deletion decreased anxiety-like behavior in mice (85). As cytokines produced by these cells can reach neurons, so can antigens from the brain reach the meninges and their resident immune cells. Therefore, the CSF constantly flows through the brain parenchyma being propelled into the brain *via* arterial pulsation through aquaporin 4 (AQP4) water channels present in the astrocyte end-feet that form the glial limitans of the BBB. As a result, the CSF flow clears the parenchyma from debris and intestinal solutes along paravenous spaces into the subarachnoid space CSF (96, 97). Moreover, it is shown that the metabolites and proteins (antigens) are traveling through the meninges and *via* lymphatic vessels into the deep cervical lymph nodes where they might be able to undergo antigen presentation (98). In fact, there is an increasing body of evidence pointing to a contribution of lymphocytes primed to self-antigens in CNS recovery and regeneration after injury and stroke (99–101). In contrast, partial, but not total depletion of Foxp3+ regulatory T cells (Tregs) is neuroprotective in a model of cerebral injury, while boosting Treg function was also shown to be beneficial in a model of stroke (102–104). Examining signatures for T cell activation and residency in aged and young mice revealed no obvious changes in CD4+ T cell phenotypes between young and old dural T cells but revealed IFN-γ production by aged meningeal T cells as a potential source for age related changes in central neuron and glia function (105–107).

Aging research and studies on Alzheimer´s disease also proved an impact on the CSF flow through followed by the lymphatic drainage. Arterial pulsation as well as lymphatic flow are decreasing with age, supporting the cumulation of metabolites and debris in the brain parenchyma (108, 109). Studies could show that decreased lymphatic flow from the meninges to the draining lymph node (LN) is associated with cognitive deficits while aged mice treated with vascular endothelial growth factor (VEGF)-c, a lymphatic endothelial cell mitogen, showed enhanced meningeal lymphatic drainage resulting in improved cognitive function (110). Furthermore, there is new evidence that a reduction in CC chemokine receptor 7 (CCR7) expression by meningeal T cells in old mice is linked to an increase in CD8+ T-bet^low^ effector and CD4+FOXP3+ Treg cells and a reduction in CD4+ T-bet^high^ IFNγ producing cells. Moreover, a hematopoietic deficiency in CCR7 can induce aging-associated changes in meningeal T cells accompanied by a reduced glymphatic influx and cognitive impairment. Deletion of CCR7 in 5xFAD transgenic mice used as an Alzheimer´s disease model promotes neurovascular and microglial activation and an increase in Aβ deposition in the brain of these mice. Conversely, old mice treated with anti-CD25 antibodies (CD4+FOXP3+Treg cells are mainly CD25^high^ expressing cells and are more responsive to anti-CD25 treatment than CD4+ and CD8+ CD25 ^low^ expressing effector T cells) showed a reduced meningeal CD4+FOXP3+ Treg cell response associated with an improved cognitive function (111). It is not shown yet, if the improved cognitive phenotype could also be induced by an associated increase in CD4+ T-bet^high^ IFN-γ producing cells. Interestingly, these changes due to CCR7 depletion or the reversal through anti-CD25 treatment could only be observed in meningeal T cells and in the respective draining lymph nodes but not in peripheral blood or liver. These studies highlight the therapeutic potential of modulating immune cells in the CNS and meningeal environment to improve brain function in aging and in neurodegenerative diseases.

While meningeal immune cells take part in CNS homeostasis from the outside, microglia function as a CNS resident specialized innate immune cell. Together with glia cells like astrocytes, oligodendrocytes and ependymal cells they all are important to maintain CNS homeostasis from within the BBB. As nicely reviewed by Greenhalgh AD and collogues (112), astrocytes are important for the formation of synapses, tight metabolic coupling with neurons to support their function, rapid neurotransmitter uptake, buffering extracellular potassium, modulation of excitability and plasticity as well as for memory consolidation and generation of circadian rhythm. Microglia, are quite important for neuronal development and learning processes due to synaptic pruning, BDNF production, phagocytosis of cellular and protein debris (e.g. Aβ) and are continuously surveilling the CNS tissue (113). They are highly reactive to changes in the brain through production of signaling molecules that can contribute to homeostasis but also contribute to disease (113). The remaining immune cells besides microglia make about 20% of all immune cells in the brain and consist of neutrophils, monocytes, dendritic cells, innate lymphoid cells and lymphocyte subsets. Under steady state conditions, the CNS environment is mostly anti-inflammatory due to predominant immune-regulatory cytokines like IL-10 and Transforming growth factor beta (TGF-β) (114, 115). This can change rapidly, as CNS immune cells, and astrocytes express PRRs to detect PAMPs and DAMPs recognizing infiltrating pathogens (116, 117) but also misfolded proteins like Aβ (52) followed by a pro-inflammatory cytokine release of IL-1β due to inflammasome activation through NLRs, that is followed by secondary secretion of cytokines like IL-6 and tumor necrosis factor (TNF)-β while IL-18 induces production of IL-17 (118, 119). This group of cellular sensors, belonging to the Nucleotide-binding oligomerization domain (NOD) -like receptor (NLR) and Nucleotide-binding oligomerization domain, Leucine rich Repeat and Pyrin domain containing)NLRP PRRs sensing PAMPs, DAMPs and other signs of active inflammation initiate the formation of so called inflammasome multiprotein complexes. There are 6 multi-protein complexes: LRR, NACHT, PYD/CARD domain containing proteins -NLRP1,3,6,12, NLRC4 and AIM2. The most studied and important inflammasome for sensing endogenous stress through signaling events such as potassium ion efflux, changes in calcium signaling and the production of reactive oxygen species by mitochondria, lysosomal leakage and cathepsin B release is the before mentioned NLRP3 (120, 121). Once activated, the NLRP3 builds a multi-protein complex by recruiting caspase 1 *via* caspase activation and recruitment domain (CARD) assembly to promote caspase enzymatic function in cleaving pro IL-1β and pro IL-18 into their active forms (122). Furthermore, it cleaves the pore forming pro-gasdermin D into its active form that induces inflammatory cell death called pyroptosis (123). The pore formed by gasdermin D promotes the release of IL-18 and IL-1β a proinflammatory cytokine that regulates the function of immune -and neuronal cells (124). Indeed, the majorly important function of inflammasomes is the detection and protection from microbes and the following IL-1β release. Therefore, the cell has to be primed in a first step through a TLR agonist like LPS or inflammatory mediators like TNF, the second signal can be provided by DAMPs or microbe associated virulence factors like toxins. There is not much known about the regulatory signals that can control inflammasome assembly and prevent pyroptosis. It is assumed that adaptive immune cells, namely CD4 T cells and memory T cells are able to suppress NLRP1 and NLRP3 mediated IL-1β secretion and capsase-1 activity, leading to the hypothesis, that the inflammasome is supposed to be active during the first days of innate immune defense against pathogens and be stopped when the adaptive immune cells are at place (125). However, there is a growing body of evidence that chronic inflammasome activity is associated with inflammatory and neurodegenerative diseases (126). High levels of these inflammasome associated cytokines decrease the homeostatic functions of astrocytes (127, 128) and microglia including an increase in glutamate release by astrocytes that is associated with excitotoxicity and microglial activation and state change to a chronic pro-inflammatory state accompanied by inflammasome activation, further promoting neurotoxicity (129–131). Inhibition of the NLRP3 inflammasome is able to convey microglia to a neuroprotective, phagocyting phenotype in a mouse model of Alzheimer´s disease (53). Indeed, a chronic state of immune suppression and anti-inflammation due to aging associated senescence of immune cells is associated with neurodegenerative diseases. There is evidence, that ER stress might contribute to the maintenance of this immune suppressive environment (132). Conversely, there are studies reporting ER stress to be highly associated with neuro-inflammation and neurodegenerative diseases (133–137), while mild ER stress can also be protective and prime CNS immune cells to better cope with infection and tissue damage (138). This paradox occurs due to the fact that the ER stress response, including activation of UPR and ERAD are important in the fight against misfolded and aggregated proteins that are hallmarks in neurodegenerative diseases like Alzheimer’s´ disease and Parkinson´s disease, but the pathways they induce are also supporting inflammation and cell death (139, 140). The clinical outcome of therapeutic targeting ER stress members *via* suppression or activation can thus both be beneficial (139).

## ER-Stress and the PNS

In contrast to the central nervous system, peripheral sensory neurons are not isolated from blood *via* a BBB, but instead are even required to sense the current metabolites, ions, proteins and chemicals in the blood and in the tissue. This is necessary to initiate adjustments *via* the autonomic nervous system including sympathetic and parasympathetic neurons but also *via* the enteric nervous system. Furthermore, sensation *via* visceral and somatic sensory neurons can be consciously sensed and therefore be important for the initiation of active behavior and responses to stimuli. Unfortunately, injury or endogenous harm of sensory neurons is therefore accompanied by dysesthesia, including paresthesia, numbness and pain. Because of their location throughout the body through innervation of organs, muscles, and skin, sensory neurons are easily exposed to external physical injuries, but they can also be easily harmed by metabolites and chemicals in the blood that have neurotoxic properties. Therefore, the most common cause for peripheral neuropathy is diabetes mellitus (141, 142). In addition, toxicity induced by alcohol consumption or chemotherapy are also common causes (143, 144). Indeed, in the periphery, neurons are imbedded in a tissue environment that is composed of Schwann cells, the peripheral equivalent to oligodendrocytes that are important for the formation of myelin sheets around the neuronal axons for faster conduction velocity, satellite cells that surround the sensory neurons soma, fibroblasts that are important for the formation of the endoneurium, perineurium and epineurium in peripheral nerves, endothelial cells from blood vessels and multiple types of immune cells including macrophages, granulocytes and lymphocytes. Thus, peripheral neuropathies can also be induced by pathologies of the surrounding cells, especially Schwann cells, that are important for the rapid signal transduction in highly myelinated cells including motor and sensory neurons. Auto-immune diseases with auto- antibodies against swan cell structures (like myelin) or structures of neuronal cells can induce acute and chronic peripheral neuropathies (145). There is high evidence, that ER stress activation occurs trough nerve injury where it contributes to neuropathic pain (146–151), while the ER stress response including UPR are required and important for neuronal regeneration and growth (152, 153). Other studies could show the involvement of ER stress in chemotherapy induced neuropathy, for example through treatment with proteasome inhibitors like Bortezomib (154) or through Paclitaxel that induces oscillatory changes in cytosolic calcium by increasing the open probability of the InsP_3_R and activating calpain, a calcium-dependent protease (155, 156). Neuropathic symptoms could be shown to be prevented or be reversed through treatment with lithium (155) or anti-oxidative plant based compounds like quercetin and lycopene that lead to an increase in neuronal growth factors like brain-derived neurotrophic factor (BDNF) and neural cell adhesion molecule (NCAM) while decreasing the expression of ER stress mediator proteins like ATF6, GRP78, PERK and IRE1 (157, 158). Furthermore, expression of the ER stress marker CCAAT/enhancer-binding protein homologous protein (CHOP) in neuropathy upon treatment with paclitaxel targeting the mitotic spindle could be inhibited *via* treatment with aucubin, a secondary plant product acting as an anti-inflammatory agent (159). More studies are reporting the contribution of ER stress in the pathology of diabetic neuropathy (160–163) and deliver possible treatment strategies by targeting the ER stress response associated inflammation (164).

Interestingly, there are also positive reports on how the UPR is involved in metabolic fates of tissues innervated by either serotonergic or dopaminergic neurons. Serotonin is thereby inducing chaperone induction and influences protein homeostasis while dopamine is important for metabolic ER-remodeling and lipid depletion (165). Activation and overexpression of the lipophagy component ehbp-1 was further sufficient to remodel ER and promote life span in a model of *Caenorhabditis elegans* (166). While peripheral neurons are therefore able to influence the ER and UPR in tissues and cells they innervate, ER stress and UPR signaling in neurons and associated Schwann cells have a major impact on neuronal functionality.

## Therapeutic Strategies

As outlined in this review ER stress, UPR and ISR are all necessary to balance cell homeostasis in the central and peripheral nervous system especially upon the defense against pathogens but also to react upon intrinsic cell stressors. Chronic activation and dysregulation of these pathways are contributing to the development of neuroinflammation and neurodegeneration. Possible treatment strategies are focusing on the reduction of stress inducers like anti-oxidative therapy and calcium signaling regulation as mentioned above, but also on restoring the balance of proteasomal degradation and autophagy or proteasome activation and activation or inhibition of translation through phosphorylation/dephosphorylation of eIF2a. Polyamines like spermidine as well as the United States Food and Drug Administration (FDA) approved anti-epileptic treatment carbamazepine are known to induce autophagy and proteasomal activation and could be shown to be neuroprotective in preclinical studies including amyotrophic lateral sclerosis (ALS) and Alzheimer´s disease (167–171). Proteasomal activation is important to provide the cell with the necessary amino acids for protein synthesis and lack of amino acids can thus induce ER stress. Therefore, the regulation of proteasome activation as well as the regulation of translation are key to a balanced protein homeostasis. The eIF2a phosphatase 1 inhibitor guanabenz is one example of how decreased global translation can be neuroprotective in the context of neurodegenerative diseases like prion disease (71) and ALS (72). In **Table 1** we list relevant treatments that further target ER stress, UPR, ISR or the Inflammasome and are already investigated in clinical trials for neurological diseases.

## Conclusion and Outlook

The ER stress response including UPR and ERAD signaling are important for the maintenance of neuronal function itself, but also for host defense and tissue immunity (172). Chronic activation of ER associated inflammation in glia cells, immune cells and neurons can turn the valued, protective function into a devastating one. Targeting of ER stress, inflammasome activity and inflammation can therefore be affective through activation and suppression, depending on the disease and progression state (120, 173, 174).

## Author Contributions

CP and EK conceptualized the work, wrote parts, reviewed and edited. CP additionally wrote major parts of the manuscript and designed the figures, tables, and screened literature. Both authors have read and agreed to the published version of the manuscript.

## Funding

This work was supported by the German Research Foundation RTG 2719 to EK and DFG PE2864/3-1 to CP. We acknowledge support for the Article Processing Charge by the German Research Foundation and the Open Access Publication Fund of the University of Greifswald.

## Conflict of Interest

The authors declare that the research was conducted in the absence of any commercial or financial relationships that could be construed as a potential conflict of interest.

## Publisher’s Note

All claims expressed in this article are solely those of the authors and do not necessarily represent those of their affiliated organizations, or those of the publisher, the editors and the reviewers. Any product that may be evaluated in this article, or claim that may be made by its manufacturer, is not guaranteed or endorsed by the publisher.
